# Deep neural network-based robotic visual servoing for satellite target tracking

**DOI:** 10.3389/frobt.2024.1469315

**Published:** 2024-10-08

**Authors:** Shayan Ghiasvand, Wen-Fang Xie, Abolfazl Mohebbi

**Affiliations:** ^1^ Department of Mechanical, Industrial and Aerospace Engineering, Concordia University, Montréal, QC, Canada; ^2^ Department of Mechanical Engineering, Polytechnique Montréal, Montréal, QC, Canada

**Keywords:** visual servoing, robot vision systems, deep neural networks, deep learning, pose estimation

## Abstract

In response to the costly and error-prone manual satellite tracking on the International Space Station (ISS), this paper presents a deep neural network (DNN)-based robotic visual servoing solution to the automated tracking operation. This innovative approach directly addresses the critical issue of motion decoupling, which poses a significant challenge in current image moment-based visual servoing. The proposed method uses DNNs to estimate the manipulator’s pose, resulting in a significant reduction of coupling effects, which enhances control performance and increases tracking precision. Real-time experimental tests are carried out using a 6-DOF Denso manipulator equipped with an RGB camera and an object, mimicking the targeting pin. The test results demonstrate a 32.04% reduction in pose error and a 21.67% improvement in velocity precision compared to conventional methods. These findings demonstrate that the method has the potential to improve efficiency and accuracy significantly in satellite target tracking and capturing.

## 1 Introduction

**FIGURE 1:**
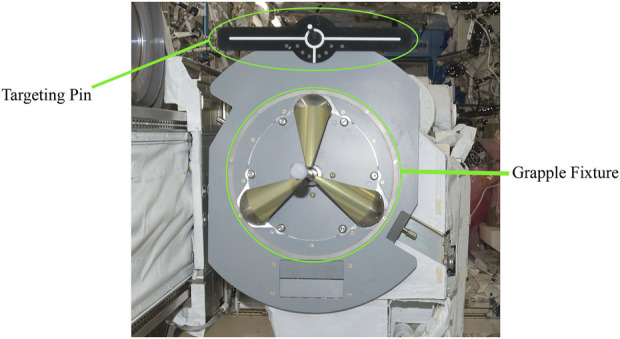
The grapple fixture and the 3D targeting pin on a servicing satellite^2^.

In spite of the technological advancements of the International Space Station (ISS), capturing incoming satellites using Canadarm2 relies heavily on manual operations. This process involves a complex interaction with the grapple fixture (Figure 1), designed for secure connection with the Canadarm2^1^. Astronauts, leveraging their training and visual cues, manually align and operate the robotic arm to successfully capture and berth these satellites.

The manual process is highly dependent on the skill and operation precision of the astronauts. Human error, inherent in any manual operation, poses significant risks in the high-stakes environment of space. Misalignment, even minor ones, can lead to mission-critical failures, jeopardizing expensive equipment and the overall success of the operation. Furthermore, the extensive training and resources required for astronauts to perform these tasks represent a significant financial and logistical investment.

Automation has proven to be an important response to the aforementioned risks associated with the manual satellite capture processes. Unlike conventional methods that rely on detailed pose information for the precise control of robot end-effectors (EE), such as kinematic modelling (Jafarinasab et al., 2019), and trajectory planning (Herrera-Aguilar and Sidobre, 2006), Image-based Visual Servoing (IBVS) method demonstrated its efficacy by obviating the need for a prior knowledge of poses. This characteristic of IBVS is particularly advantageous because it avoids the tedious task of pose estimation. Another noteworthy aspect of IBVS is its eye-in-hand configuration, a configuration that mirrors the existing setup on the Canadarm2 (Chang and Evans, 2009). This setup is compatible with the operational requirements of capturing satellites, where the capturing device must adjust its position and orientation in real-time based on the visual input from the target satellite. Furthermore, IBVS has been acknowledged for its robust performance in unstructured environments (Ahlin et al., 2016). The unpredictable and dynamically changing nature of space, with no structured environment, requires a flexible and adaptive approach such as IBVS. For instance, Shi et al. (2012) proposed a visual servoing approach (switching between IBVS and position-based visual servoing (PBVS)) for a space robot to capture a cooperative target. However, this approach is limited by its requirement for binocular vision, which makes it unsuitable for the Canadarm2 equipped with one camera. In addition, the low frame rate of four frames per second (FPS) presented in their work may reduce the accuracy required for successful target tracking.

In IBVS, the selection of an effective set of image features (s) is vital for controlling the motions in robot’s degrees of freedoms (DOF). Image features correspond to the projection of a physical feature of some object onto the camera image plane (Corke et al., 1996). The relationship between the change of a set of image features over time $\left({\dot{s}}^{k\times 1}\right)$ and the camera velocity $\left(v_{c}^{6\times 1}\right)$ is given by Equation 1 (Chaumette and Hutchinson, 2006):

$$\dot{s}=L_{s}v_{c}$$ (1)

The matrix $L_{s}$ of dimensions $R^{k\times 6}$ is referred to as the interaction matrix associated with the feature vector s (Chaumette and Hutchinson, 2006).

The commonly used image features are the coordinates of points, straight lines or ellipses in the image plane. However, they are restricted to a limited set of objects (Khiabani et al., 2019), and they may easily get out of the field of view (FOV) during servoing, and losing any of the features would cause a failure in the visual servoing. To tackle these issues, several researchers have proposed to use image moments derived from the regions of the image (Huang et al., 2022; Shaw et al., 2016; Li et al., 2015; Zhou et al., 2021), allowing for the representation of arbitrary object shapes (He et al., 2019). It is worth noting that an ideal image feature would associate uniquely with the motion in a single DOF, leading to minimal interference among the motions in other DOFs. In other terms, the interaction matrix derived from the ideal image features will be an identity matrix (He et al., 2019).

Nevertheless, as highlighted in both Tahri and Chaumette (2005) and Chaumette (2004), it is challenging to achieve an ideal interaction matrix (identity matrix) due to inherent nonlinearities in Equation 3. To solve this problem, researchers have sought two kinds of image features to achieve decoupling among the 6 DOFs, (i) Analytical function-based image features (Huang et al., 2022; Wu et al., 2018; Liu et al., 2009) and (ii) Data-driven features (Quaccia et al., 2024; Zhou et al., 2021; Zhao et al., 2012).

In the analytical function-based image features, the objective is to create an analytical function corresponding to a motion in specific DOF. These analytical functions are derived from image moments and are ideally invariant to other DOFs, so they can accurately represent their corresponding specific motion. The pioneering work in this area by Chaumette (2004) presented an analytical basis for image feature functions. Chaumette’s approach used the object’s centroid to infer x and y positions, its area for depth z , two innovative functions for β and γ based on Hu’s invariants (Hu, 1962), and the object’s orientation for α . However, the proposed set of image features, while corresponding the movement in a single degree of freedom of the end effector, unintentionally produced unnecessary movements in other degrees, which is referred to as coupling. For example, the image moments within β and γ DOFs suffered from intrinsic coupling, resulting in an ineffective control in practice. Furthermore, the proposed orientation features were shape-dependent ( $S_{x}$ and $S_{y}$ for symmetric and $P_{x}$ and $P_{y}$ for asymmetric objects), which limits the generalizability of this approach.

Subsequent studies have attempted to resolve these couplings. For instance, Tahri and Chaumette (2005) used normalization techniques to mitigate the coupling effects within the translational DOFs. However, fully decoupled features remained unattainable. In the search for shape-independent rotational features, Tamtsia et al. (2013) proposed the features based on shifted moments with invariant properties for both symmetric and asymmetric objects. Although their approach was robust to some extent, it did not fully solve the decoupling problem between β and γ .

The research work by Liu et al. (2009) further decoupled the problematic rotational features and performed well in practice but lacked generalizability as it distinctively proposed separate features for small and large objects. Recent studies, including those by Huang et al. (2022), Khiabani et al. (2019), and He et al. (2019), have continued using the analytical image feature functions. Nonetheless, despite their applicability, the challenge of complete decoupling remains unsolved. Furthermore, these improved features are subject to limitations when confronted with a variety of object sizes and shapes.

Leveraging the machine learning techniques and the universal approximation capabilities of neural networks, several studies proposed data-driven features. Machine learning methods such as support vector machine (SVM) is proposed by Li et al. (2015) to learn the mapping model from four moment invariants to two virtual moments in order to decouple β and γ motions.

In addition, Neural Network (NN)-based methods have demonstrated promising results in this area. Zhao et al. (2012) and Zhou et al. (2021) proposed a method using shallow neural networks to identify two rotational decoupled image features about the *x* and *y*-axes ( β and γ ). However, the method’s sole reliance on these two features resulted in an incomplete decoupling, leaving non-zero elements in the interaction matrices of other degrees of freedom (DOFs). This lack of decoupling potentially introduces undesirable rotational velocities, $v_{\beta }$ and $v_{\gamma }$ , which affects the control in these axes. In addition, the study’s data set was severely limited, consisting of only a narrow set of data points from a fixed position. This lack of diversity undermines the model’s applicability across the manipulator’s workspace, limiting its effectiveness beyond the specific training conditions. Furthermore, Liu and Li (2019) designed a convolution neural network (CNN) to estimate parameters such as x , y , z , and α directly from images. Nevertheless, it encounters a significant challenge in terms of computing complexity. The computational intensity required to compute the output significantly slows the processing per control loop. This delay could potentially result in a longer sampling time, which may introduce sluggish control response, and hence decrease the precision and effectiveness of the robot manipulation.

This study proposes a set of decoupled image features specifically tailored to the unique geometry of the targeting pin used in satellite capturing. By achieving a near-diagonal interaction matrix, we aim to minimize the coupling effects, enhancing the accuracy and efficiency of the target tracking. This decoupling is crucial for smooth and precise operations, reducing the risk of errors and improving overall system performance.

Our chosen method to identify and optimize these decoupled features involves Deep Neural Network (DNN) training. DNNs offer a sophisticated approach to model complex relationships and patterns, making them ideal for extracting and refining the necessary image features for effective visual servoing. Through extensive training and optimization, we aim to develop a robust DNN model capable of estimating the pose of the robot for the closed-loop visual servoing. The experimental results on a Denso robot show that the developed DNN-based visual servoing can accurately guide the manipulator to track the targeting pin in real-time. The developed method is expected to enhance the precision, safety, and efficiency of space operations on the ISS.

This paper presents our research on DNN-based visual servoing for satellite target tracking. Sections 2.1–2.4 explore the core of our method, detailing image feature definition, hyperparameter tuning, and the architecture of the DNN model. Section 2.5 describes the generation of a comprehensive dataset, which is essential for training the DNN model. Sections 3, 4 present a series of practical tests and validations that demonstrate the effectiveness of our approach. The paper concludes with Section 5, which summarizes our findings, their significance for space robotics, and potential directions for future research.

## 2 Materials and methods

### 2.1 DNN-based visual servoing

The choice of the set of visual features for decoupling the 6 DOF motion has been a well-known challenge in visual servoing. The commonly used point features introduce a non-diagonal interaction matrix which usually contains the terms that involve the depth of the point (Z), the image coordinate (x, y), and partial derivatives of the projection equations. Using the appropriate combination of image moments to estimate the pose may result in good decoupling and linearizing properties. The power of a deep learning-based approach is leveraged to propose a set of image features based on various image moments that are almost perfectly decoupled for the specific geometry of the targeting pin (see Figure 1). This section starts with the feature definition and estimation approach and proceeds to the detailed architecture of the proposed DNN model and fine-tuning the hyperparameters of the model.

### 2.2 Image feature definition

Consider a 6-DOF manipulator with a camera installed on its end-effector. The target object is assumed to be stationary with respect to the robot’s reference frame. We choose the different combinations of image moments as the input to the DNN model to estimate the pose. The set of image features of the target object is represented as Equation 2:

$$s=\left[\begin{array}{c} c_{x}x\\ c_{y}y\\ c_{z}z\\ c_{\beta }\beta \\ c_{\gamma }\gamma \\ c_{\alpha }\alpha \end{array}\right],$$ (2)

where $c_{x}$ , $c_{y}$ , $c_{z}$ , $c_{\beta }$ , $c_{\gamma }$ , and $c_{\alpha }$ are realized through DNN models. When we take the derivative of the above feature with respect to time, we would like to obtain a diagonal interaction matrix $L_{s}$ which relates the set of image features to the velocity vector $v_{c}$ :

$$\dot{s}=L_{s}v_{c},$$ (3)

where $L_{s}$ and $v_{c}$ are defined in Equation 4:

$$\begin{array}{cc} L_{s} & =\mathrm{diag}\left(c_{x},c_{y},c_{z},c_{\beta },c_{\gamma },c_{\alpha }\right),\\ v_{c} & =\left[\dot{x},\dot{y},\dot{z},\dot{\beta },\dot{\gamma },\dot{\alpha }\right]. \end{array}$$ (4)

It is noticed that Equation 3 is obtained under the conditions that DNN models representing $c_{x}$ , $c_{y}$ , $c_{z}$ , $c_{\beta }$ , $c_{\gamma }$ , $c_{\alpha }$ are time invariant and independent from each other, which poses challenge on training DNN to realize. However, under ideal conditions where the camera pose is precisely estimated, the interaction matrix becomes the identity matrix (see Equation 5).

$$L_{s}=I_{6}.$$ (5)

Deep neural networks (DNNs) prove to be a robust approach for this type of estimation. In this study, the proposed network’s input is a set of moments, central moments, and a few engineered features and the output aims to predict the camera’s six-dimensional (6D) pose. Its specific architecture will be discussed in the next subsection.

### 2.3 Architecture

The DNN-based visual servoing approach has an architecture designed to estimate the camera’s pose effectively with respect to the targeting pin. Designing an optimal DNN architecture is an iterative process that requires extensive evaluations in different scenarios to identify the architecture that best solves the problem. The initial network used an architecture with shared neurons to estimate both the rotational and translational poses of the camera. However, experimental results showed that the complexity of translational poses required a deeper network, while rotational poses could be successfully obtained from a shallower network. It is worth noting that experimenting with deeper networks resulted in overfitting during training for rotational poses.

**FIGURE 2:**
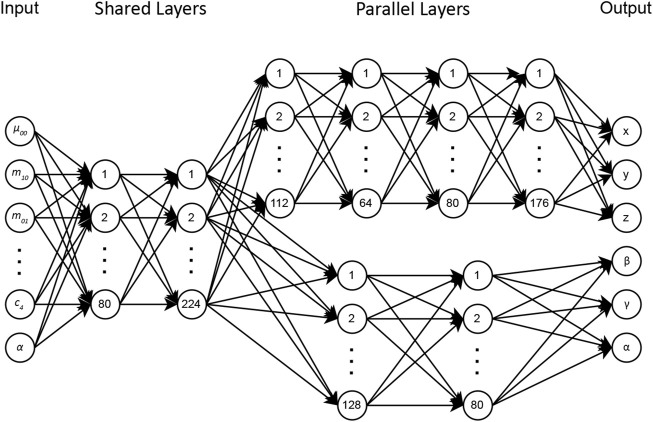
DNN architecture for camera pose estimation from image moments.

Translational and orientational poses differ fundamentally, requiring unique approaches for their accurate estimation. Thus, we proposed an architecture in which the initial two layers are shared for rotational and translational DOFs, while the subsequent layers operate in parallel. For translational elements (x,y,z) , this network consists of six hidden layers, with node distributions of 80, 224, 112, 64, 80, and 176. For the rotational elements (β,γ,α) , four hidden layers are employed with distributions of 80, 224, 128, and 80 nodes. Figure 2 illustrates the proposed architecture.

The model’s input includes the image moments up to the third order $\left(\mu_{00},m_{10},m_{01},\mu_{11},\mu_{20},\mu_{02},\mu_{21},\mu_{12},\mu_{30},\mu_{03}\right)$ and five additional engineered features. These engineered features encompass four invariants $\left(c_{1},c_{2},c_{3},c_{4}\right)$ , derived from moment invariants as suggested by Tahri and Chaumette (2005), and α . These engineered features enhance the rotational degrees of freedom estimations, due to the invariance of $c_{1},c_{2},c_{3}$ , and $c_{4}$ to 2D translation and 2D rotation, as well as the correlation between α $\left(\alpha =\frac{1}{2}\arctan\left(\frac{2\mu_{11}}{\mu_{20}-\mu_{02}}\right)\right)$ and the $R_{z}$ component. $c_{1}$ through $c_{4}$ are defined in Equation 6:

$$\left\{\begin{array}{cc} I_{1} & \;=-\mu_{20}\mu_{02}+\mu_{11}^{2}\\ I_{2} & \;={\left(\mu_{20}-\mu_{02}\right)}^{2}+4\mu_{11}^{2}\\ I_{3} & \;={\left(\mu_{30}-3\mu_{12}\right)}^{2}+{\left(3\mu_{21}-\mu_{03}\right)}^{2}\\ I_{4} & \;={\left(\mu_{30}+\mu_{12}\right)}^{2}+{\left(\mu_{21}+\mu_{03}\right)}^{2}\\ I_{5} & \;=-\mu_{30}^{2}\mu_{03}^{2}+6\mu_{30}\mu_{21}^{3}-4\mu_{30}\mu_{12}^{3}-4\mu_{21}^{3}\mu_{03}+3\mu_{21}^{2}\mu_{12}^{2}\\ I_{6} & \;=3\mu_{30}^{2}\mu_{12}^{2}+2\mu_{30}^{2}\mu_{03}^{2}-6\mu_{30}\mu_{21}^{2}\mu_{12}-6\mu_{30}\mu_{21}\mu_{12}\mu_{03}\\ & \;+2\mu_{30}\mu_{12}^{3}+3\mu_{21}^{4}+2\mu_{21}^{3}\mu_{03}+3\mu_{21}^{2}\mu_{03}^{2}-12\mu_{21}\mu_{12}^{2}\mu_{03}+6\mu_{12}^{4}\\ I_{7} & \;=-\mu_{30}^{3}\mu_{03}+3\mu_{30}^{2}\mu_{21}\mu_{12}-2\mu_{30}\mu_{21}^{3}-3\mu_{30}\mu_{21}^{2}\mu_{03}\\ \; & \;+6\mu_{30}\mu_{21}\mu_{12}^{2}+3\mu_{30}\mu_{12}^{2}\mu_{03}+\mu_{30}\mu_{03}^{3}-3\mu_{21}^{3}\mu_{12}\\ \; & \;-6\mu_{21}^{2}\mu_{12}\mu_{03}+3\mu_{21}\mu_{12}^{3}-3\mu_{21}\mu_{12}\mu_{03}^{2}+2\mu_{12}^{3}\mu_{03} \end{array}\right.\left\{\begin{array}{cc} c_{1} & \;=\frac{I_{1}}{I_{2}}\\ c_{2} & \;=\frac{I_{3}}{I_{4}}\\ c_{3} & \;=\frac{I_{5}}{I_{6}}\\ c_{4} & \;=\frac{I_{7}}{I_{6}} \end{array}\right.$$ (6)

By employing this architecture, we aim to achieve high accuracy in both translational and rotational pose predictions.

### 2.4 Hyperparameter tuning

In the search for optimal model performance, we explored several hyperparameters:

• Activation Functions: These are mathematical expressions that determine the output of a node in our network. We considered various options, including ‘Relu’, ‘Leaky Relu’, ‘Tanh’, and ‘Sigmoid’.

• Batch Size: This refers to the number of training examples used in one iteration. We explored a range from 32 to 512.

• Learning Rate: This hyperparameter determines the step size at each iteration while moving towards a minimum of the loss function. We considered values of $10^{-2}$ , $10^{-3}$ , and $10^{-4}$ .

• Optimizers: These are algorithms or methods used to adjust model parameters to minimize the model error. We looked into two options: Adam and Adamw (Adam weight decay).

Given the vast hyperparameter space, an efficient strategy of Random Search was used to circumvent the computational cost associated with exhaustively exploring every combination. Random Search samples a fixed number of hyperparameter combinations from the total pool to balance computational efficiency and the broadness of exploration, and increase the probability of finding a near-optimal set of hyperparameters.

Table 1 summarizes the hyperparameter values that were found to be most effective, and were used to initialize the training.

**TABLE 1:** Optimal hyperparameter values from random search.

Hyperparameter	Optimal value
Activation Function	ReLU
Batch Size	512
Learning Rate	$1\times 10^{-3}$
Optimizer	Adam

### 2.5 Data set generation

**FIGURE 3:**
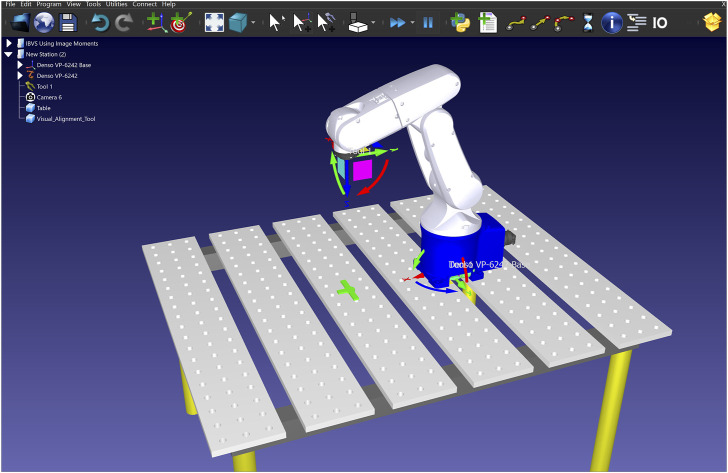
RoboDK environment including the Denso robot, the camera, and the targeting pin.

In a supervised approach, training is the most critical part, and to fully unleash the power of the deep learning model, we need a high-quality training dataset. To this end, we created a dataset which consists of both synthetic and real-world images from the targeting pin. For generating the synthetic data, we used RoboDK, a sophisticated offline programming and simulation platform designed specifically for robotics applications. This section presents a novel approach that overcomes a significant limitation in the data generation process that ensures the targeting pin remains in the image plane when random positions are assigned to the camera. As depicted in Figure 3, the simulation setup consisted of a Denso manipulator equipped with a camera mimicking the properties of our real-world setup.

In this approach, the goal is to randomly assign values to all 6 DOFs with the constraint that the object is in the image plane. Considering this constraint, first, the x, y, and z coordinates of the camera (mounted on the end effector) were randomly generated within the working range of the manipulator. Next, to generate the orientational DOFs randomly, the camera is supposed to initially have the object at the center of the image plane. This pose was computed using a “Look at” function. The “Look at” function is typically designed to orient the camera towards a specific point (object’s centroid in our case) in the environment.

This function starts by defining a source point (camera) and a target point (object), along with initial vectors for up (U), front (F), and right (V). V, U and F are initially considered as the unit vectors pointing in the positive *x*, *y* and *z*-axes, respectively (see Equation 7).

$$V=\left[\begin{array}{c} 1\\ 0\\ 0 \end{array}\right],U=\left[\begin{array}{c} 0\\ 1\\ 0 \end{array}\right],F=\left[\begin{array}{c} 0\\ 0\\ 1 \end{array}\right].$$ (7)

The first step is to calculate the new front vector $F^{\prime }$ , which points from the source to the target. This vector is obtained by subtracting the source position $\left(x_{c}\right)$ from the target position $\left(x_{o}\right)$ and normalizing the resulting vector (Equation 8):

$$F^{\prime }=\frac{x_{o}-x_{c}}{\left\|x_{o}-x_{c}\right\|}.$$ (8)

Next, we calculate the new up vector $U^{\prime }$ . We start by subtracting the projection of U onto $F^{\prime }$ from U and then normalize it (Equation 9):

$$U^{\prime }=\frac{U-\left(U\cdot F^{\prime }\right)F^{\prime }}{\left\|U-\left(U\cdot F^{\prime }\right)F^{\prime }\right\|}.$$ (9)

In case the resulting vector has zero magnitude, we default $U^{\prime }$ to be the same as the original front vector F $\left(U^{\prime }=F\right)$ .

The third axis, $V^{\prime }$ , is calculated as the cross product of $U^{\prime }$ and $F^{\prime }$ (Equation 10):

$$V^{\prime }=U^{\prime }\times F^{\prime }.$$ (10)

These new basis vectors ( $V^{\prime }$ , $U^{\prime }$ , $F^{\prime }$ ) form the rotation matrix for the new camera pose (Equation 11):

Roc=V′U′F′. (11)

Finally, the pose of the camera is represented as a 4×4 transformation matrix (Equation 12):

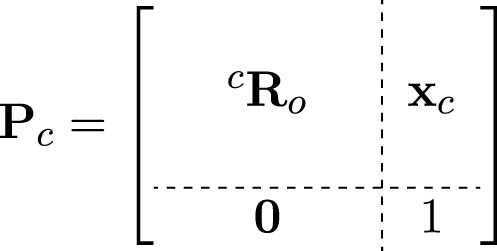

(12)



Next, to randomly generate rotational DOFs, we first rotate the camera about its optical axis within a predefined range. Rotating about the optical center will not result in losing the object from the image plane. Then we determine the rotation bounds for the camera about its *x* and *y*-axes, based on the distance of the camera from the object. We can obtain the rotation limits by performing linear interpolation between the predefined bounds at two known distances, ensuring that the object remains in the image plane. Consequently, the camera was rotated around its *x* and *y*-axes for a random value within these limits. Table 2 includes the boundaries used for the pose parameters.

**TABLE 2:** Ranges of the pose parameters ( ${\text{Limit}}_{\beta }$ and ${\text{Limit}}_{\gamma }$ are determined by linear interpolation).

Parameter	Minimum value	Maximum value
X	207.5 (mm)	407.5 (mm)
Y	−150 (mm)	150 (mm)
Z	150 (mm)	500 (mm)
β	$-{\text{Limit}}_{\beta }$	${\text{Limit}}_{\beta }$
γ	$-{\text{Limit}}_{\gamma }$	${\text{Limit}}_{\gamma }$
α	45°	135°

The dataset includes the entries for the calculated image moments and central moments of the captured image, while the corresponding camera pose is collected as the label. Initially, in the simulated environment, 434,528 random poses were generated within the ranges of Table 2, sequentially commanded to the Denso manipulator. At each pose, an image was captured by the mounted camera and processed into a binary representation, and the moments and central moments were then computed.

In addition to the synthetic data, we also captured real data to enrich the data set and enhance the robustness of our model in real-world scenarios. To realize this, we used a similar approach to generate random camera poses. However, due to the slower operational tempo of the physical setup compared to the simulator, we recorded data during the motion of the end effector from one random pose to another. However, to ensure that only valid data is captured, it is necessary to apply constraints that exclude images that do not fully capture the targeting pin. As a result, to ensure the presence of the object, the data points were only recorded when a single contour larger than 500 pixels was detected in the image and when the bounding box of the target pin was at least 10 pixels away from the image borders. This cautious approach resulted in 1912 distinct poses commanded to the Denso robot, yielding a total of 198,588 valid real-world data points (including data points captured during the camera movement from one random pose to the other).

To provide a visual representation of how the data set is created, two videos were prepared to show the process in action. The first video demonstrates the simulation environment data generation, accessible at this link, while the second video shows the real environment data generation, available at this link.

**FIGURE 4:**
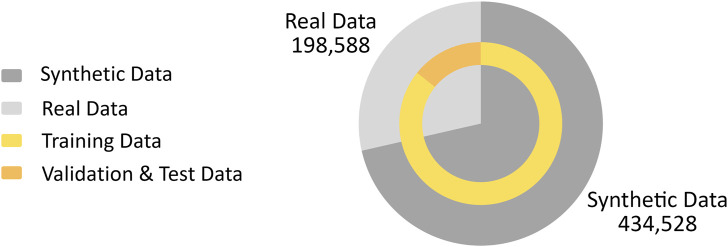
Distribution of synthetic and real data used for training, validation, and testing.

The final data set was carefully split, with all synthetic and half of the real data allocated for training. The remaining real data was evenly divided between the validation and test sets (Figure 4). This approach originated from our experimental findings that relying only on either synthetic or real data reduced the performance on the test set, likely due to the real environment’s noise and lighting conditions and the limited diversity of poses in the real data. As a result of combining both data sources, we were able to achieve a balance that captured both the complexity of real-world scenarios and provided enough variability for robust model training.

## 3 Results

First, in Section 3.1, we introduce the experimental setup mimicking the satellite target tracking in the space. Section 3.2 presents the initial performance evaluation of our trained DNN model for pose estimation. In Section 3.3 we explore the computation of the interaction matrix and present experimental results on target tracking with different initial poses.

### 3.1 Experimental setup

**FIGURE 5:**
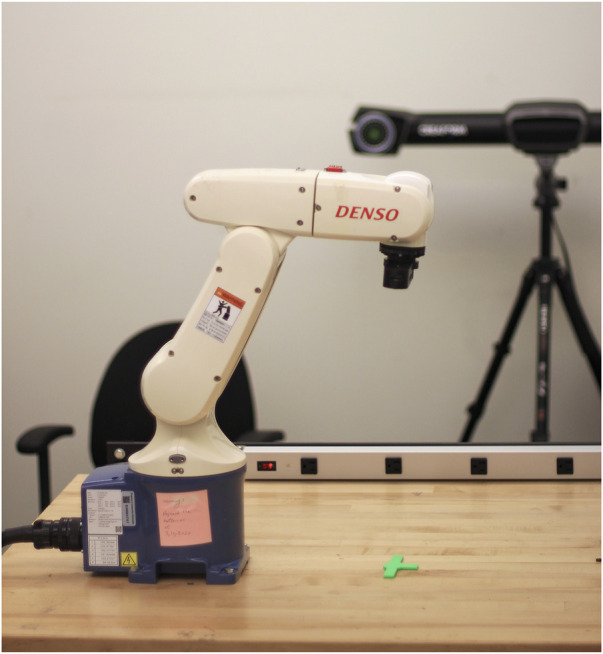
Experimental setup within the real environment.

Similar to the setup in our simulations (see Figure 3), our experimental layout includes a 6-DOF Denso robotic manipulator, an Intel RealSense D415 RGB camera for high-quality image capture, and a green 3D-printed targeting pin that mimics the ISS’s guiding markers, as illustrated in Figure 5.

### 3.2 Training results

The DNN model, as described in Section 2.3, was trained for 1,000 epochs, where the loss value eventually plateaued, indicating an optimal learning point. To determine the pose estimation accuracy of the model, we used the Mean Absolute Error (MAE) along with a Scaled MAE metric customized for our multi-output scenario. The scaled MAE was necessary due to the different units and magnitudes of the outputs (translational values in millimeters and rotational values in degrees). To compute it, we first normalized each output’s MAE by its range from Table 3, ensuring uniform error scaling across all outputs.

**TABLE 3:** Ranges for output elements of the dataset.

Element	x (mm)	y (mm)	z (mm)	β°	γ°	α°
Minimum	157.5	−150	150	129.91	−55.03	−117.36
Maximum	411.54	150	500	240.5	56.65	129.11
Range	254.04	300	350	110.59	111.68	246.47

The ‘best’ model was selected based on its performance on the validation set. Table 4 presents the MAE data for this model, offering insights into its translational and rotational pose accuracy.

**TABLE 4:** Final model’s mean absolute error data.

Element	MAE	Average MAE	Scaled MAE	Average scaled MAE
x	7.45 (mm)	5.82 (mm)	$2.93\times 10^{-2}$	$1.54\times 10^{-2}$
y	5.83 (mm)		$1.94\times 10^{-2}$	
z	4.18 (mm)		$1.20\times 10^{-2}$	
β	1.32°	1.37°	$1.19\times 10^{-2}$	
γ	1.78°		$1.59\times 10^{-2}$	
α	1.02°		$0.41\times 10^{-2}$	

### 3.3 Experiments

It is necessary to derive an interaction matrix for the final DNN model. While our initial aim was to derive a diagonal interaction matrix, the practical limitations in achieving zero-error pose estimation necessitated the use of the actual interaction matrix in our experiments. In Equation 3, the 6×6 interaction matrix represents how the DNN model’s predicted image features correlate with the manipulator’s the motion in six axes. For every data point in the test set, the model predicted six image features. The elements in the interaction matrix represent the slopes of the linear regression lines, each comparing a predicted image feature against every actual degree of freedom. This approach helps us understand the impact of each actual movement on the predicted features.

$$L_{s_{\mathrm{DNN}}}=\left[\begin{array}{cccccc} 0.94 & 0.03 & -0.12 & -0.16 & -3.24 & 0.03\\ 0.05 & 0.98 & 0 & -3.57 & -0.12 & -0.22\\ -0.21 & -0.01 & 0.99 & -0.15 & 0.53 & -0.16\\ -0.02 & -0.18 & -0.01 & 0.97 & 0.05 & 0.04\\ -0.14 & 0 & 0.01 & 0.01 & 0.93 & 0\\ 0 & -0.04 & -0.02 & 0.16 & -0.02 & 0.99 \end{array}\right]$$ (13)

As evident from the interaction matrix in Equation 13, the diagonal elements $L_{s_{\mathrm{DNN}}}\left[i,i\right]$ (where i ranges from 1 to 6) are very close to 1, while the non-diagonal elements are close to zero, which aligns with our objective. However, the noticeable exceptions are the elements $L_{s_{\mathrm{DNN}}}\left[1,5\right]=-3.24$ and $L_{s_{\mathrm{DNN}}}\left[2,4\right]=-3.57$ . These values indicate a correlation between the x prediction of the DNN during $R_{y}$ movement and the y prediction during $R_{x}$ movement. This correlation is understandable, as rotations around the x $\left(R_{x}\right)$ and y $\left(R_{y}\right)$ axes in the manipulator’s frame cause corresponding movements along the *y* and *x*-axes in the image plane. Additionally, the elements $L_{s_{\mathrm{DNN}}}\left[4,2\right]=-0.18$ and $L_{s_{\mathrm{DNN}}}\left[5,1\right]=-0.14$ in the fourth and fifth rows are higher than other non-diagonal elements, emphasizing the ‘x and $R_{y}$ ’ and ‘y and $R_{x}$ ’ interconnections in the final DNN model. Improving the DNN’s accuracy in the estimations can further address these interconnections.

We tested the model with five distinct initial poses, ensuring a mix of positive and negative initial errors for each degree of freedom. The chosen initial poses, labelled A through E, are detailed in Table 5.

**TABLE 5:** Initial and desired poses.

	Pose					
	x (mm)	y (mm)	z (mm)	β (deg)	γ (deg)	α (deg)
A	314.05	37.05	413.32	166.21	−11.40	−16.25
B	308.93	−57.99	434.48	200.75	−8.26	10.25
C	368.71	−74.09	386.80	200.14	−3.59	12.00
D	257.79	−27.34	495.22	196.62	6.91	22.33
E	276.98	51.01	249.78	163.99	15.68	16.01
Desired	307.5	0	300	180	0	0

**FIGURE 6:**
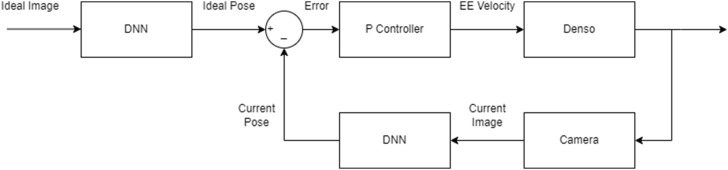
DNN-based visual servoing block diagram.

**FIGURE 7:**
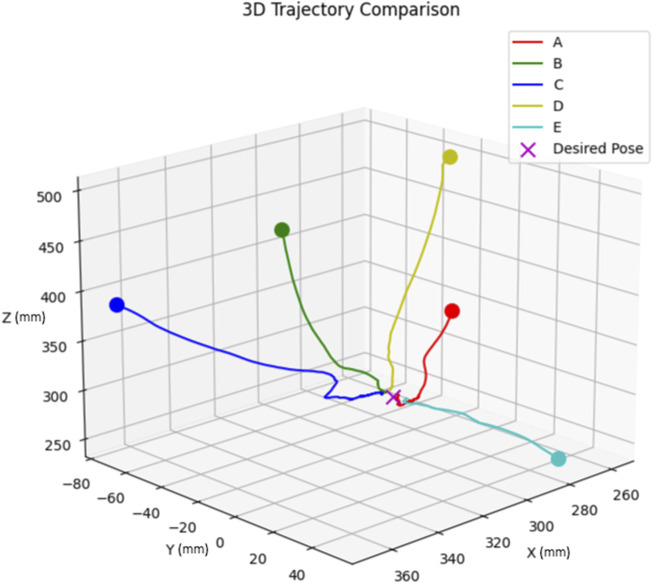
Trajectory comparison of five different initial poses.

The block diagram of the DNN-based visual servoing is depicted in Figure 6, where we used a proportional controller and the DNN extracts feature (pose) from the images. For these tests, we adjusted the P controller for each degree of freedom to ensure the manipulator’s end effector converges within 1 cm and 3° to the desired pose. The resulting trajectories for each initial pose are depicted in Figure 7.

As shown from Figure 7, the end effector follows an almost straight path from its start to the target. However, in practice, as the end effector gets close to the desired pose, we noted minor shakiness in its movement. This is caused by small oscillations in the pose estimates, which are the outputs of the neural network.

## 4 Discussion

To validate the proposed features derived from the DNN method, some comparisons were made with a prominent set of features in the literature. This set consists of Tahri and Chaumette (2005)’s features, which are the centroid coordinates $x_{g}$ and $y_{g}$ , the area a , and the rotation α . Additionaly, for rotations about the *x* and *y*-axes, Liu et al. (2009)’s features ( $s_{x}$ and $s_{y}$ as described in Equation 14) are used. From now on, the combination of these features is referred to as the Liu method $\left(\left[x_{g},y_{g},a,s_{x},s_{y},\alpha \right]\right)$ .

$$\left\{\begin{array}{c} x_{g}=\frac{m_{10}}{m_{00}}\;\\ y_{g}=\frac{m_{01}}{m_{00}}\;\\ a=m_{00}\;\\ s_{x}=0.1-\left(c_{1}c_{2}+s_{1}s_{2}\right)/I_{3}^{\left(9/4\right)}\;\\ s_{y}=\left(s_{1}c_{2}-c_{1}s_{2}\right)/I_{3}^{\left(9/4\right)}\;\\ \alpha =\frac{1}{2}\arctan\left(\frac{2\mu_{11}}{\mu_{20}-\mu_{02}}\right),\; \end{array}\right.$$ (14)

where $m_{ij}$ and $\mu_{pq}$ are moments of order i+j and central moments of order p+q , respectively. Also, c_1_, c_2_, s_1_, and s_2_ are defined in Equation 15:

$$\left\{\begin{array}{c} c_{1}=\mu_{20}-\mu_{02}\;\\ c_{2}=\mu_{03}-3\mu_{21}\;\\ s_{1}=2\mu_{11}\;\\ s_{2}=\mu_{30}-3\mu_{12}\; \end{array}\right..$$ (15)

The Liu method’s features have the units of $\left[px,px,px^{2},px^{\frac{10}{9}},px^{\frac{10}{9}},rad\right]$ . In contrast, the DNN method’s features, which represent pose ([x,y,z,β,γ,α]) , have units of [mm,mm,mm,deg,deg,deg] . Because of these unit differences, each method needs its own set of controller gains. To ensure a fair comparison, the P controllers were carefully adjusted for each method, aiming for convergence within 1 cm for translational movements and 3° for rotational ones. The tuned gains of P controllers can be seen in Table 6.

**TABLE 6:** Tuned P controllers for the DNN and Liu methods.

Method	Controller gains					
	$K_{x}$	$K_{y}$	$K_{z}$	$K_{\beta }$	$K_{\gamma }$	$K_{\alpha }$
DNN	0.09	0.09	0.12	0.003	0.003	0.003
Liu	0.9	0.9	150	30	60	0.3

**FIGURE 8:**
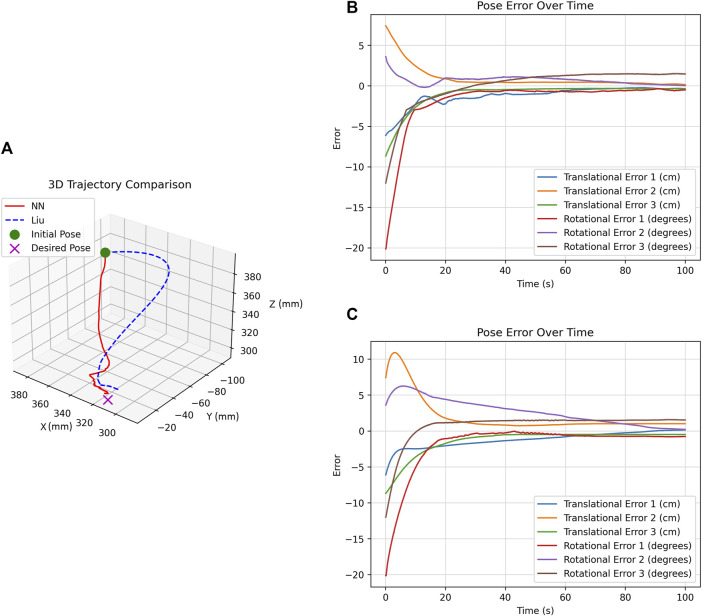
Analysis of DNN and Liu Methods for initial pose C **(A)** Trajectory comparison. **(B)** DNN method’s pose error over time. **(C)** Liu method’s pose error over time.

Running the methods for 100 s under different initial poses yielded similar results. Therefore, to illustrate the comparison, we present the results for initial pose C (as outlined in Table 5) as an example. Figure 8A shows the trajectory plot, comparing the end-effector path for both the Liu and DNN methods from initial pose C. The plots clearly show that the DNN method achieves a direct and efficient trajectory from the starting pose to the target. In contrast, the Liu method resulted in a curved, less efficient path. It is important to highlight that the Liu method operates on feature error, not pose error. As a result, in certain experiments, the end effector stopped close to the desired pose due to minimal feature differences between images. However, the DNN method almost consistently identified these differences, ending up at the correct pose.

Figures 8B, C show the pose error of the DNN and Liu methods over time, respectively. A closer look at these plots reveals that the DNN method has a faster response with less overshoot, which is desirable for our application.

While Figure 8 shows improvements in the performance, it does not provide the quantitative details needed for a comprehensive analysis. Thus, we use three metrics: RMS (Root Mean Square), Max (Maximum value), and STD (Standard Deviation).

• RMS: This metric measures the overall oscillation intensity, whether in terms of pose error or velocity. A high RMS value in the pose error indicates deviations from the desired pose, and when observed in velocity, it points to speed fluctuations.

• Max: Serving as a measure for extremes, the Max metric identifies the largest positional deviation or the most significant speed variation.

• STD: It shows the variability of the pose error or velocity around its mean value. High STD values emphasize inconsistencies.

For an organized overview, Table 7 lists these metrics over the same experiments with initial pose C (as outlined in Table 5).

**TABLE 7:** Metrics comparison for Initial Pose C.

Data	Element	Method	RMS	Max	STD
Pose Error	x	Liu	16.156	−61.210	10.394
		DNN	17.234	−61.210	12.007
	y	Liu	34.253	109.058	26.731
		DNN	16.534	74.092	13.674
	z	Liu	22.790	−86.790	17.954
		DNN	18.610	−86.790	15.570
	β	Liu	4.125	−20.152	3.625
		DNN	3.673	−20.152	3.215
	γ	Liu	3.284	6.247	1.784
		DNN	1.033	3.592	0.546
	α	Liu	2.266	−12.009	2.146
		DNN	2.332	−12.009	2.332
Velocity	x	Liu	3.666	28.843	3.619
		DNN	1.740	−5.675	1.634
	y	Liu	2.131	17.032	2.021
		DNN	1.517	6.226	1.367
	z	Liu	1.902	6.403	1.679
		DNN	2.166	−9.765	1.986
	β	Liu	0.004	−0.031	0.004
		DNN	0.011	−0.050	0.010
	γ	Liu	0.009	0.047	0.009
		DNN	0.002	−0.018	0.002
	α	Liu	0.009	−0.041	0.008
		DNN	0.007	0.033	0.006

From the detailed analysis of Table 7, it is evident that the DNN method’s performance significantly improved for most of the degrees of freedom. The consistently lower RMS, Max, and STD values indicate a more stable and predictable performance. However, there are notable exceptions in the x and α poses where the DNN method shows a marginally worse performance. Interestingly, when we focus on velocity, the DNN method compensates for both of the aforementioned pose errors. For velocities, it is worth noting that the DNN method’s performance metrics for the z and γ directions are higher, indicating a more variable or unpredictable movement.

To better compare the performance of the methods and to evaluate the improvement of the DNN method, the average RMS values for pose error and velocity are presented in Table 8. It is evident that the Deep Neural Network (DNN) method substantially outperforms the Liu method in several key aspects. The DNN method reduced the average RMS values for translational pose error and velocity by over 47%, demonstrating a robust capability in improving the system’s responsiveness and accuracy. Despite these gains, the DNN method shows a smaller improvement of 13.85% in rotational pose error and a slight decrease in performance for rotational velocities. This small decrease is on the order of $10^{-3}$ , which makes it negligible.

**TABLE 8:** comparison of average RMS values and improvements in translational and rotational DOFs for pose error and velocity.

Category	Parameter	Liu	DNN	Improvement (%)	Avg. Improvement (%)
Pose Error	Translational (mm)	26.075	12.980	50.22	32.04
	Rotational (deg)	3.600	3.101	13.85	
Velocity	Translational (mm/s)	2.473	1.294	47.69	21.67
	Rotational (deg/s)	7.67 $\times 10^{-3}$	8 $\times 10^{-3}$	−4.35	

The DNN-based visual servoing method’s adaptability to unanticipated scenarios is demonstrated in this video, showing the manipulator’s response when the targeting pin is arbitrarily re-positioned in the workspace. The video highlights the system’s capability to efficiently track the targeting pin, ensuring it remains centered and parallel in the camera’s view within a short amount of time.

## 5 Conclusion

This research addresses the challenge of coupling in visual servoing to autonomously track the targeting pin on servicing satellites using a robotic manipulator. In this paper, we presented a novel deep learning-based visual servoing approach that uses image moments to precisely estimate the camera’s pose to achieve decoupled image features. The main contributions and conclusions of this research are as follows:

• Development of DNN-based Visual Servoing: A parallelized DNN architecture for estimating the camera’s pose is meticulously designed. These pose elements are treated as a novel set of decoupled image features, offering a nearly diagonal interaction matrix.

• Data set generation: We have implemented a data generation strategy that combines synthetic and real data. While 6D poses were randomly generated, an innovative strategy ensures that the object remains in the image. This comprehensive training dataset covers a broad spectrum of scenarios, ensuring the DNN model is well-prepared to handle real-world conditions effectively.

• Comparative Analysis with Established Techniques: A comprehensive experimental validation of the neural network approach is conducted, demonstrating significant improvements in trajectory, pose accuracy, and velocity of the end effector compared to established visual servoing techniques.

The most important impact of this study is its adaptability for controlling various robotic manipulators in marker-based applications. By using our training procedure for any targeting pin, one can potentially achieve performances outperforming some classical image moment-based visual servoing methods.

## 6 Future works

The following suggestions can potentially improve the proposed methods’ performance and generalizability:

• Dataset Enhancement: Creating a dataset that uses the real targeting pin (Figure 1) or ensuring that the dataset’s environment closely resembles space lighting conditions can improve the accuracy of pose predictions.

• Canadarm2 Kinematics: Investigate the application of the proposed methods by testing or simulating on the Canadarm2 kinematics.

• Hyperparameter Refinement: Continuous tuning and experimentation with the network’s architecture and hyperparameters can improve performance.

• Transfer Learning: Using insights from established pre-trained pose estimation models and adapting them to the current problem might yield better results.

• Network Ensembling: Aggregating outputs from diverse network architectures can enhance accuracy, as different models might specialize in recognizing distinct features.

• Direct Image Input: Utilizing the image itself (rather than its moments) as the network’s input could provide insights potentially overlooked when solely relying on image moments.

## Data Availability

The datasets presented in this article are not readily available because the dataset can be only accessible for the robotic engineers. Requests to access the datasets should be directed to SG, shayan.gh@hotmail.com.

## Footnotes

1
https://bit.ly/47PzoHV

2
http://iss.jaxa.jp/library/photo/iss022e020034.php

## References

[B1] Ahlin K. Joffe B. Hu A.-P. McMurray G. Sadegh N. (2016). Autonomous leaf picking using deep learning and visual-servoing. IFAC-PapersOnLine 49, 177–183. 5th IFAC Conference on Sensing, Control and Automation Technologies for Agriculture AGRICONTROL 2016. 14–17 August 2016, Seattle, WA, USA. 10.1016/j.ifacol.2016.10.033

[B2] Chang V. Evans L. (2009). “Chapter 9 - robotic systems safety,” in Safety design for space systems. Editors Musgrave G. E. Larsen A. S. M. Sgobba T. (Burlington: Butterworth-Heinemann), 301–318. 10.1016/B978-0-7506-8580-1.00009-9

[B3] Chaumette F. (2004). Image moments: a general and useful set of features for visual servoing. IEEE Trans. Robotics 20, 713–723. 10.1109/TRO.2004.829463

[B4] Chaumette F. Hutchinson S. (2006). Visual servo control. i. basic approaches. IEEE Robotics and Automation Mag. 13, 82–90. 10.1109/MRA.2006.250573

[B5] Corke P. (1996). Visual Control of Robots: high-performance visual servoing. United Kingdom: Research Studies Press Taunton.

[B6] He Z. Wu C. Zhang S. Zhao X. (2019). Moment-based 2.5-d visual servoing for textureless planar part grasping. IEEE Trans. Industrial Electron. 66, 7821–7830. 10.1109/TIE.2018.2886783

[B7] Herrera-Aguilar I. Sidobre D. (2006). “Soft motion trajectory planning and control for service manipulator robot,” in Workshop on physical human-robot interaction in anthropic domains at IROS, 13–22.

[B8] Hu M.-K. (1962). Visual pattern recognition by moment invariants. IRE Trans. Inf. theory 8, 179–187. 10.1109/TIT.1962.1057692

[B9] Huang Y. Li J. Zhang X. Xie K. Li J. Liu Y. (2022). A surgeon preference-guided autonomous instrument tracking method with a robotic flexible endoscope based on dvrk platform. IEEE Robotics Automation Lett. 7, 2250–2257. 10.1109/LRA.2022.3143305

[B10] Jafarinasab M. Sirouspour S. Dyer E. (2019). Model-based motion control of a robotic manipulator with a flying multirotor base. IEEE/ASME Trans. Mechatronics 24, 2328–2340. 10.1109/TMECH.2019.2936760

[B11] Khiabani P. M. Ramezanzadeh J. Taghirad H. D. (2019). “Implementation of an improved moment-based visual servoing controller on an industrial robot,” in 2019 7th international conference on robotics and mechatronics (ICRoM), 125–131. 10.1109/ICRoM48714.2019.9071911

[B12] Li W. Ye G. Wan H. Zheng S. Lu Z. (2015). “Decoupled control for visual servoing with svm-based virtual moments,” in 2015 IEEE International Conference on Information and Automation, 08-10 August 2015, Lijiang, China: IEEE, 2121–2126. 10.1109/ICInfA.2015.7279638

[B13] Liu J. Li Y. (2019). An image based visual servo approach with deep learning for robotic manipulation. CoRR, 07727. 10.48550/arXiv.1909.07727

[B14] Liu S. Xie W.-F. Su C.-Y. (2009). “Image-based visual servoing using improved image moments,” in 2009 international conference on information and automation, 22-24 June 2009, Zhuhai, Macau: IEEE, 577–582.

[B15] Quaccia M. André A. Yoshiyasu Y. Caron G. (2024). “A study on learned feature maps toward direct visual servoing,” in 16th IEEE/SICE international symposium on system integration (SII), 08-11 January 2024, Ha Long, Vietnam: IEEE and SICE.

[B16] Shaw Q. Hu J. Fang Y. Liu W. Qi J. Zhu G.-N. (2016). “Image moments based visual servoing of robot using an adaptive controller,” in 2016 Asia-Pacific Conference on Intelligent Robot Systems (ACIRS), 20-22 July 2016, Tokyo, Japan: IEEE, 57–61. 10.1109/ACIRS.2016.7556188

[B17] Shi Y. Liang B. Wang X. Xu W. Liu H. (2012). “Study on intelligent visual servoing of space robot for cooperative target capturing,” in 2012 IEEE International Conference on Information and Automation, 06-08 June 2012, Shenyang, China, IEEE, 733–738. 10.1109/ICInfA.2012.6246915

[B18] Tahri O. Chaumette F. (2005). Point-based and region-based image moments for visual servoing of planar objects. IEEE Trans. Robotics 21, 1116–1127. 10.1109/TRO.2005.853500

[B19] Tamtsia A. Y. Tahri O. Mezouar Y. Djalo H. Tonye E. (2013). “New results in images moments-based visual servoing,” in 2013 IEEE International Conference on Robotics and Automation, 06-10 May 2013, Karlsruhe, Germany: IEEE, 5271–5276.

[B20] Wu D. Zhong X. Zhang X. Peng X. Zou C. (2018). “Uncalibrated image-based visual servoing based on joint space and image moment,” in 2018 37th Chinese control conference (CCC), 25-27 July 2018, Wuhan, China: IEEE, 5391–5397. 10.23919/ChiCC.2018.8483954

[B21] Zhao Y. Xie W. Wang T. (2012). “Neural network-based image moments for visual servoing of planar objects,” in 2012 IEEE/ASME international conference on advanced intelligent mechatronics (AIM), 11-14 July 2012, Kaohsiung, Taiwan: IEEE, 268–273.

[B22] Zhou Y. Zhang Y. Gao J. An X. (2021). “Visual servo control of underwater vehicles based on image moments,” in 2021 6th IEEE international conference on advanced robotics and mechatronics (ICARM), 03-05 July 2021, Chongqing, China: IEEE, 899–904. 10.1109/ICARM52023.2021.9536071

